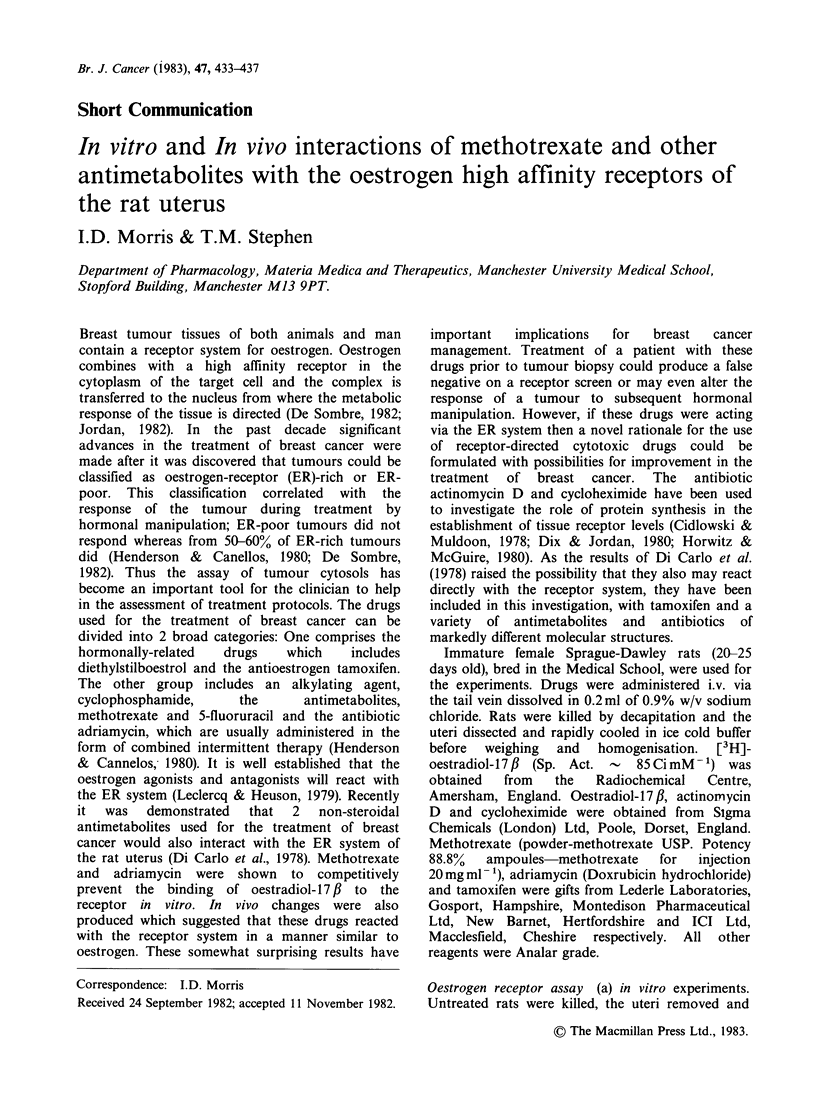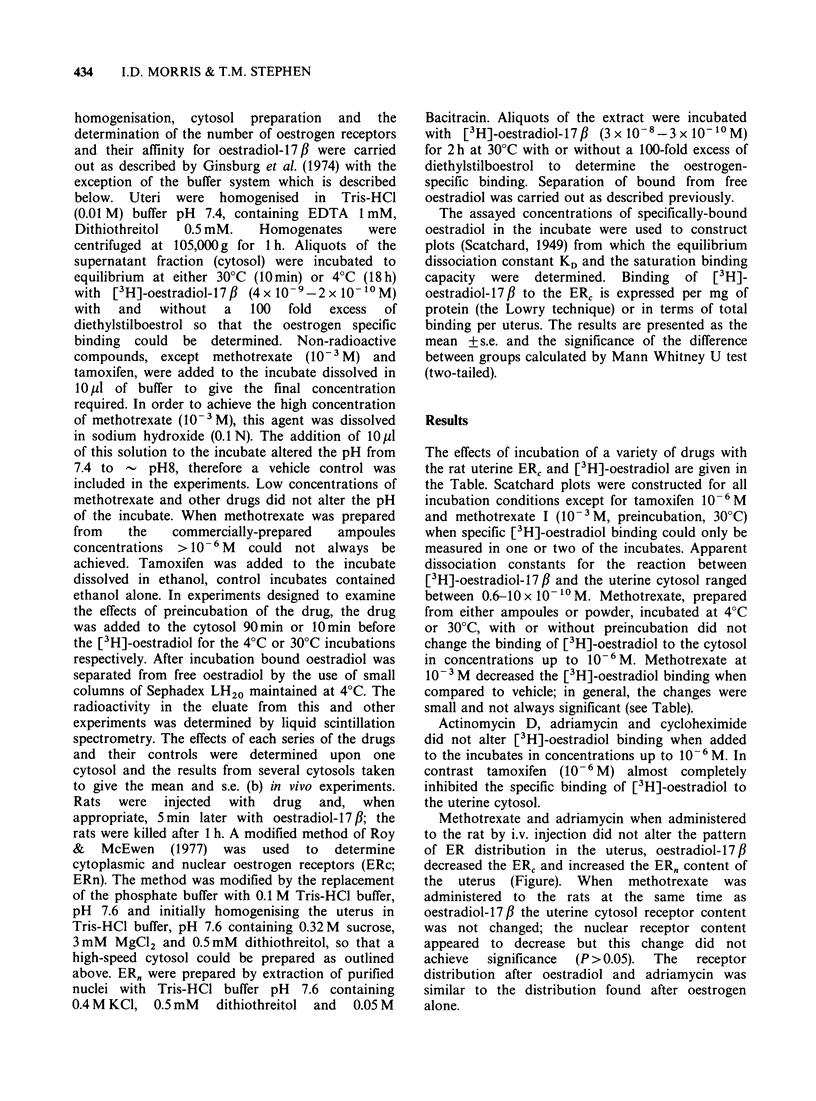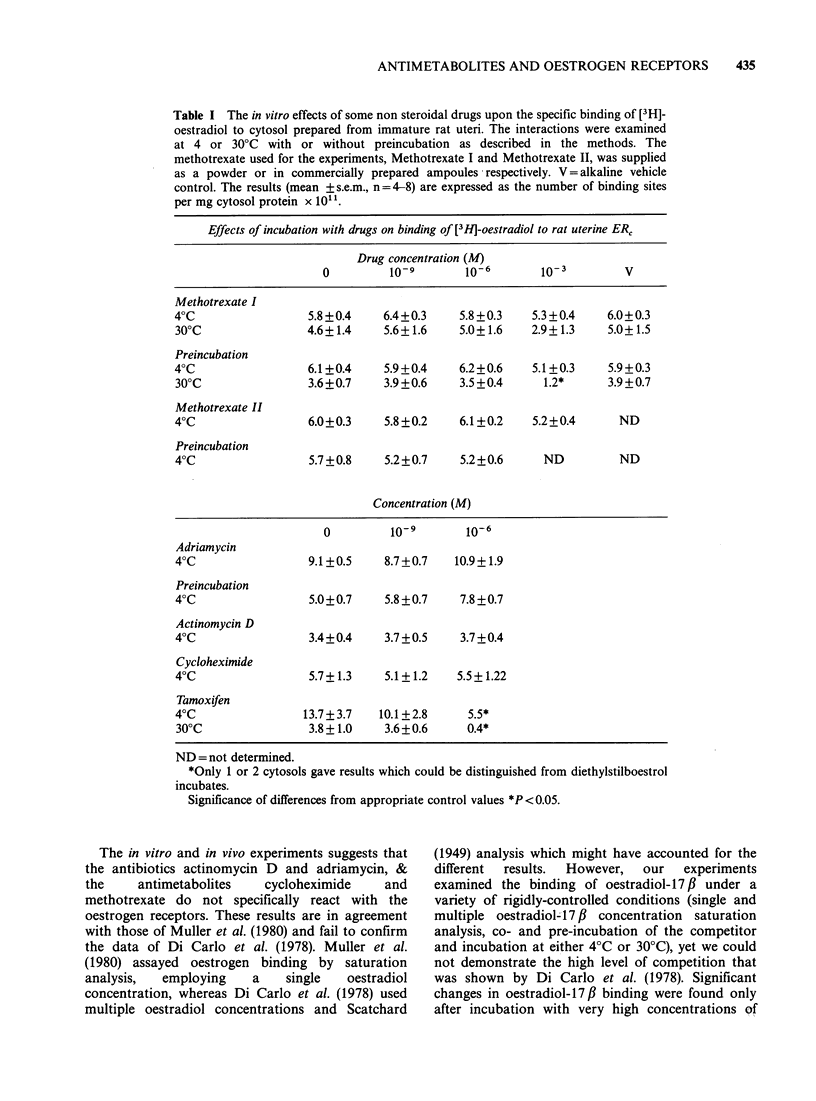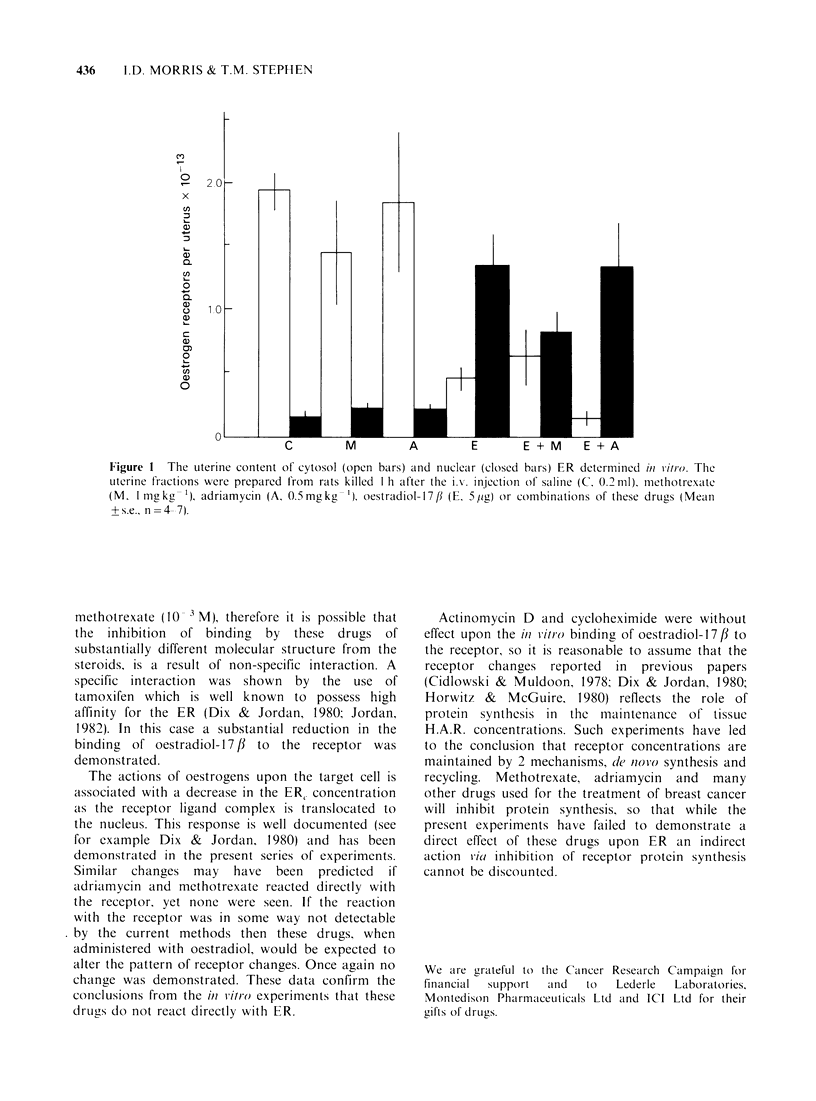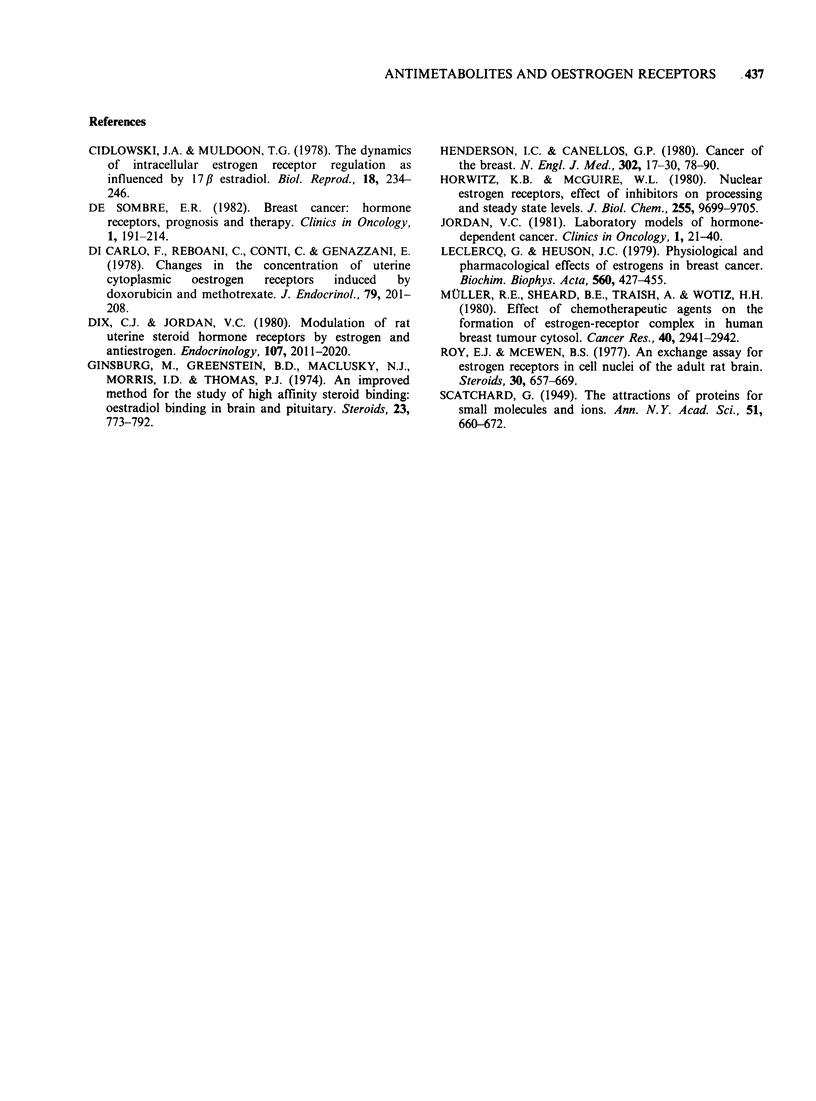# In vitro and in vivo interactions of methotrexate and other antimetabolites with the oestrogen high affinity receptors of the rat uterus.

**DOI:** 10.1038/bjc.1983.66

**Published:** 1983-03

**Authors:** I. D. Morris, T. M. Stephen


*Br. J. Cancer* (1983), **47**, 433–437

### Short Communication

### *In vitro* and *In vivo* interactions of methotrexate and other antimetabolites with the oestrogen high affinity receptors of the rat uterus

I.D. Morris & T.M. Stephen

*Department of Pharmacology, Materia Medica and Therapeutics, Manchester University Medical School, Stopford Building, Manchester M13 9PT.*

Breast tumour tissues of both animals and man contain a receptor system for oestrogen. Oestrogen combines with a high affinity receptor in the cytoplasm of the target cell and the complex is transferred to the nucleus from where the metabolic response of the tissue is directed (De Sombre, 1982; Jordan, 1982). In the past decade significant advances in the treatment of breast cancer were made after it was discovered that tumours could be classified as oestrogen-receptor (ER)-rich or ER-poor. This classification correlated with the response of the tumour during treatment by hormonal manipulation; ER-poor tumours did not respond whereas from 50–60% of ER-rich tumours did (Henderson & Canellos, 1980; De Sombre, 1982). Thus the assay of tumour cytosols has become an important tool for the clinician to help in the assessment of treatment protocols. The drugs used for the treatment of breast cancer can be divided into 2 broad categories: One comprises the hormonally-related drugs which includes diethylstilboestrol and the antioestrogen tamoxifen. The other group includes an alkylating agent, cyclophosphamide, the antimetabolites, methotrexate and 5-fluoruracil and the antibiotic adriamycin, which are usually administered in the form of combined intermittent therapy (Henderson & Cannelos, 1980). It is well established that the oestrogen agonists and antagonists will react with the ER system (Leclercq & Heuson, 1979). Recently it was demonstrated that 2 non-steroidal antimetabolites used for the treatment of breast cancer would also interact with the ER system of the rat uterus (Di Carlo *et al*., 1978). Methotrexate and adriamycin were shown to competitively prevent the binding of oestradiol-17$\beta$ to the receptor *in vitro*. *In vivo* changes were also produced which suggested that these drugs reacted with the receptor system in a manner similar to oestrogen. These somewhat surprising results have important implications for breast cancer management. Treatment of a patient with these drugs prior to tumour biopsy could produce a false negative on a receptor screen or may even alter the response of a tumour to subsequent hormonal manipulation. However, if these drugs were acting via the ER system then a novel rationale for the use of receptor-directed cytotoxic drugs could be formulated with possibilities for improvement in the treatment of breast cancer. The antibiotic actinomycin D and cycloheximide have been used to investigate the role of protein synthesis in the establishment of tissue receptor levels (Cidlowski & Muldoon, 1978; Dix & Jordan, 1980; Horwitz & McGuire, 1980). As the results of Di Carlo *et al.* (1978) raised the possibility that they also may react directly with the receptor system, they have been included in this investigation, with tamoxifen and a variety of antimetabolites and antibiotics of markedly different molecular structures.

Immature female Sprague-Dawley rats (20–25 days old), bred in the Medical School, were used for the experiments. Drugs were administered i.v. via the tail vein dissolved in 0.2 ml of 0.9% w/v sodium chloride. Rats were killed by decapitation and the uteri dissected and rapidly cooled in ice cold buffer before weighing and homogenisation. [$^3$H]-oestradiol-17$\beta$ (Sp. Act. $\sim$ 85 Ci mM$^{-1}$) was obtained from the Radiochemical Centre, Amersham, England. Oestradiol-17$\beta$, actinomycin D and cycloheximide were obtained from Sigma Chemicals (London) Ltd, Poole, Dorset, England. Methotrexate (powder-methotrexate USP. Potency 88.8% ampoules—methotrexate for injection 20 mg ml$^{-1}$), adriamycin (Doxrubicin hydrochloride) and tamoxifen were gifts from Lederle Laboratories, Gosport, Hampshire, Montedison Pharmaceutical Ltd, New Barnet, Hertfordshire and ICI Ltd, Macclesfield, Cheshire respectively. All other reagents were Analar grade.

*Oestrogen receptor assay* (a) *in vitro* experiments. Untreated rats were killed, the uteri removed and

Correspondence: I.D. Morris

Received 24 September 1982; accepted 11 November 1982.

© The Macmillan Press Ltd., 1983.

homogenisation, cytosol preparation and the determination of the number of oestrogen receptors and their affinity for oestradiol-17$\beta$ were carried out as described by Ginsburg *et al.* (1974) with the exception of the buffer system which is described below. Uteri were homogenised in Tris-HCl (0.01 M) buffer pH 7.4, containing EDTA 1 mM, Dithiothreitol 0.5 mM. Homogenates were centrifuged at 105,000 g for 1 h. Aliquots of the supernatant fraction (cytosol) were incubated to equilibrium at either 30°C (10 min) or 4°C (18 h) with [$^3$H]-oestradiol-17$\beta$ ($4 \times 10^{-9} - 2 \times 10^{-10}$ M) with and without a 100 fold excess of diethylstilboestrol so that the oestrogen specific binding could be determined. Non-radioactive compounds, except methotrexate ($10^{-3}$ M) and tamoxifen, were added to the incubate dissolved in 10 $\mu$l of buffer to give the final concentration required. In order to achieve the high concentration of methotrexate ($10^{-3}$ M), this agent was dissolved in sodium hydroxide (0.1 N). The addition of 10 $\mu$l of this solution to the incubate altered the pH from 7.4 to ~ pH8, therefore a vehicle control was included in the experiments. Low concentrations of methotrexate and other drugs did not alter the pH of the incubate. When methotrexate was prepared from the commercially-prepared ampoules concentrations $>10^{-6}$ M could not always be achieved. Tamoxifen was added to the incubate dissolved in ethanol, control incubates contained ethanol alone. In experiments designed to examine the effects of preincubation of the drug, the drug was added to the cytosol 90 min or 10 min before the [$^3$H]-oestradiol for the 4°C or 30°C incubations respectively. After incubation bound oestradiol was separated from free oestradiol by the use of small columns of Sephadex $LH_{20}$ maintained at 4°C. The radioactivity in the eluate from this and other experiments was determined by liquid scintillation spectrometry. The effects of each series of the drugs and their controls were determined upon one cytosol and the results from several cytosols taken to give the mean and s.e. (b) *in vivo* experiments. Rats were injected with drug and, when appropriate, 5 min later with oestradiol-17$\beta$; the rats were killed after 1 h. A modified method of Roy & McEwen (1977) was used to determine cytoplasmic and nuclear oestrogen receptors (ERc; ERn). The method was modified by the replacement of the phosphate buffer with 0.1 M Tris-HCl buffer, pH 7.6 and initially homogenising the uterus in Tris-HCl buffer, pH 7.6 containing 0.32 M sucrose, 3 mM $MgCl_2$ and 0.5 mM dithiothreitol, so that a high-speed cytosol could be prepared as outlined above. $ER_n$ were prepared by extraction of purified nuclei with Tris-HCl buffer pH 7.6 containing 0.4 M KCl, 0.5 mM dithiothreitol and 0.05 M Bacitracin. Aliquots of the extract were incubated with [$^3$H]-oestradiol-17$\beta$ ($3 \times 10^{-8} - 3 \times 10^{-10}$ M) for 2 h at 30°C with or without a 100-fold excess of diethylstilboestrol to determine the oestrogen-specific binding. Separation of bound from free oestradiol was carried out as described previously.

The assayed concentrations of specifically-bound oestradiol in the incubate were used to construct plots (Scatchard, 1949) from which the equilibrium dissociation constant $K_D$ and the saturation binding capacity were determined. Binding of [$^3$H]-oestradiol-17$\beta$ to the $ER_c$ is expressed per mg of protein (the Lowry technique) or in terms of total binding per uterus. The results are presented as the mean $\pm$s.e. and the significance of the difference between groups calculated by Mann Whitney U test (two-tailed).

### Results

The effects of incubation of a variety of drugs with the rat uterine $ER_c$ and [$^3$H]-oestradiol are given in the Table. Scatchard plots were constructed for all incubation conditions except for tamoxifen $10^{-6}$ M and methotrexate I ($10^{-3}$ M, preincubation, 30°C) when specific [$^3$H]-oestradiol binding could only be measured in one or two of the incubates. Apparent dissociation constants for the reaction between [$^3$H]-oestradiol-17$\beta$ and the uterine cytosol ranged between $0.6–10 \times 10^{-10}$ M. Methotrexate, prepared from either ampoules or powder, incubated at 4°C or 30°C, with or without preincubation did not change the binding of [$^3$H]-oestradiol to the cytosol in concentrations up to $10^{-6}$ M. Methotrexate at $10^{-3}$ M decreased the [$^3$H]-oestradiol binding when compared to vehicle; in general, the changes were small and not always significant (see Table).

Actinomycin D, adriamycin and cycloheximide did not alter [$^3$H]-oestradiol binding when added to the incubates in concentrations up to $10^{-6}$ M. In contrast tamoxifen ($10^{-6}$ M) almost completely inhibited the specific binding of [$^3$H]-oestradiol to the uterine cytosol.

Methotrexate and adriamycin when administered to the rat by i.v. injection did not alter the pattern of ER distribution in the uterus, oestradiol-17$\beta$ decreased the $ER_c$ and increased the $ER_n$ content of the uterus (Figure). When methotrexate was administered to the rats at the same time as oestradiol-17$\beta$ the uterine cytosol receptor content was not changed; the nuclear receptor content appeared to decrease but this change did not achieve significance ($P>0.05$). The receptor distribution after oestradiol and adriamycin was similar to the distribution found after oestrogen alone.

**Table I** The *in vitro* effects of some non steroidal drugs upon the specific binding of [$^3$H]-oestradiol to cytosol prepared from immature rat uteri. The interactions were examined at 4 or 30°C with or without preincubation as described in the methods. The methotrexate used for the experiments, Methotrexate I and Methotrexate II, was supplied as a powder or in commercially prepared ampoules respectively. V = alkaline vehicle control. The results (mean ± s.e.m., n = 4–8) are expressed as the number of binding sites per mg cytosol protein × $10^{11}$.

*Effects of incubation with drugs on binding of* [$^3$*H*]-*oestradiol to rat uterine* $ER_c$

| | Drug concentration (*M*) | | | | |
|---|---|---|---|---|---|
| | 0 | $10^{-9}$ | $10^{-6}$ | $10^{-3}$ | V |
| *Methotrexate I* | | | | | |
| 4°C | 5.8±0.4 | 6.4±0.3 | 5.8±0.3 | 5.3±0.4 | 6.0±0.3 |
| 30°C | 4.6±1.4 | 5.6±1.6 | 5.0±1.6 | 2.9±1.3 | 5.0±1.5 |
| *Preincubation* | | | | | |
| 4°C | 6.1±0.4 | 5.9±0.4 | 6.2±0.6 | 5.1±0.3 | 5.9±0.3 |
| 30°C | 3.6±0.7 | 3.9±0.6 | 3.5±0.4 | 1.2* | 3.9±0.7 |
| *Methotrexate II* | | | | | |
| 4°C | 6.0±0.3 | 5.8±0.2 | 6.1±0.2 | 5.2±0.4 | ND |
| *Preincubation* | | | | | |
| 4°C | 5.7±0.8 | 5.2±0.7 | 5.2±0.6 | ND | ND |

| | Concentration (*M*) | | |
|---|---|---|---|
| | 0 | $10^{-9}$ | $10^{-6}$ |
| *Adriamycin* | | | |
| 4°C | 9.1±0.5 | 8.7±0.7 | 10.9±1.9 |
| *Preincubation* | | | |
| 4°C | 5.0±0.7 | 5.8±0.7 | 7.8±0.7 |
| *Actinomycin D* | | | |
| 4°C | 3.4±0.4 | 3.7±0.5 | 3.7±0.4 |
| *Cycloheximide* | | | |
| 4°C | 5.7±1.3 | 5.1±1.2 | 5.5±1.22 |
| *Tamoxifen* | | | |
| 4°C | 13.7±3.7 | 10.1±2.8 | 5.5* |
| 30°C | 3.8±1.0 | 3.6±0.6 | 0.4* |

ND = not determined.

*Only 1 or 2 cytosols gave results which could be distinguished from diethylstilboestrol incubates.

Significance of differences from appropriate control values *$P < 0.05$.

The *in vitro* and *in vivo* experiments suggests that the antibiotics actinomycin D and adriamycin, & the antimetabolites cycloheximide and methotrexate do not specifically react with the oestrogen receptors. These results are in agreement with those of Muller *et al.* (1980) and fail to confirm the data of Di Carlo *et al.* (1978). Muller *et al.* (1980) assayed oestrogen binding by saturation analysis, employing a single oestradiol concentration, whereas Di Carlo *et al.* (1978) used multiple oestradiol concentrations and Scatchard (1949) analysis which might have accounted for the different results. However, our experiments examined the binding of oestradiol-17$\beta$ under a variety of rigidly-controlled conditions (single and multiple oestradiol-17$\beta$ concentration saturation analysis, co- and pre-incubation of the competitor and incubation at either 4°C or 30°C), yet we could not demonstrate the high level of competition that was shown by Di Carlo *et al.* (1978). Significant changes in oestradiol-17$\beta$ binding were found only after incubation with very high concentrations of

**Figure 1** The uterine content of cytosol (open bars) and nuclear (closed bars) ER determined *in vitro*. The uterine fractions were prepared from rats killed 1 h after the i.v. injection of saline (C, 0.2 ml), methotrexate (M, 1 mg kg$^{-1}$), adriamycin (A, 0.5 mg kg$^{-1}$), oestradiol-17$\beta$ (E, 5 $\mu$g) or combinations of these drugs (Mean ± s.e., n = 4–7).

methotrexate ($10^{-3}$ M), therefore it is possible that the inhibition of binding by these drugs of substantially different molecular structure from the steroids, is a result of non-specific interaction. A specific interaction was shown by the use of tamoxifen which is well known to possess high affinity for the ER (Dix & Jordan, 1980; Jordan, 1982). In this case a substantial reduction in the binding of oestradiol-17$\beta$ to the receptor was demonstrated.

The actions of oestrogens upon the target cell is associated with a decrease in the $ER_c$ concentration as the receptor ligand complex is translocated to the nucleus. This response is well documented (see for example Dix & Jordan, 1980) and has been demonstrated in the present series of experiments. Similar changes may have been predicted if adriamycin and methotrexate reacted directly with the receptor, yet none were seen. If the reaction with the receptor was in some way not detectable by the current methods then these drugs, when administered with oestradiol, would be expected to alter the pattern of receptor changes. Once again no change was demonstrated. These data confirm the conclusions from the *in vitro* experiments that these drugs do not react directly with ER.

Actinomycin D and cycloheximide were without effect upon the *in vitro* binding of oestradiol-17$\beta$ to the receptor, so it is reasonable to assume that the receptor changes reported in previous papers (Cidlowski & Muldoon, 1978; Dix & Jordan, 1980; Horwitz & McGuire, 1980) reflects the role of protein synthesis in the maintenance of tissue H.A.R. concentrations. Such experiments have led to the conclusion that receptor concentrations are maintained by 2 mechanisms, *de novo* synthesis and recycling. Methotrexate, adriamycin and many other drugs used for the treatment of breast cancer will inhibit protein synthesis, so that while the present experiments have failed to demonstrate a direct effect of these drugs upon ER an indirect action *via* inhibition of receptor protein synthesis cannot be discounted.

We are grateful to the Cancer Research Campaign for financial support and to Lederle Laboratories, Montedison Pharmaceuticals Ltd and ICI Ltd for their gifts of drugs.

**References**

CIDLOWSKI, J.A. & MULDOON, T.G. (1978). The dynamics of intracellular estrogen receptor regulation as influenced by 17$\beta$ estradiol. *Biol. Reprod.*, **18**, 234–246.

DE SOMBRE, E.R. (1982). Breast cancer: hormone receptors, prognosis and therapy. *Clinics in Oncology*, **1**, 191–214.

DI CARLO, F., REBOANI, C., CONTI, C. & GENAZZANI, E. (1978). Changes in the concentration of uterine cytoplasmic oestrogen receptors induced by doxorubicin and methotrexate. *J. Endocrinol.*, **79**, 201–208.

DIX, C.J. & JORDAN, V.C. (1980). Modulation of rat uterine steroid hormone receptors by estrogen and antiestrogen. *Endocrinology*, **107**, 2011–2020.

GINSBURG, M., GREENSTEIN, B.D., MACLUSKY, N.J., MORRIS, I.D. & THOMAS, P.J. (1974). An improved method for the study of high affinity steroid binding: oestradiol binding in brain and pituitary. *Steroids*, **23**, 773–792.

HENDERSON, I.C. & CANELLOS, G.P. (1980). Cancer of the breast. *N. Engl. J. Med.*, **302**, 17–30, 78–90.

HORWITZ, K.B. & MCGUIRE, W.L. (1980). Nuclear estrogen receptors, effect of inhibitors on processing and steady state levels. *J. Biol. Chem.*, **255**, 9699–9705.

JORDAN, V.C. (1981). Laboratory models of hormone-dependent cancer. *Clinics in Oncology*, **1**, 21–40.

LECLERCQ, G. & HEUSON, J.C. (1979). Physiological and pharmacological effects of estrogens in breast cancer. *Biochim. Biophys. Acta*, **560**, 427–455.

MÜLLER, R.E., SHEARD, B.E., TRAISH, A. & WOTIZ, H.H. (1980). Effect of chemotherapeutic agents on the formation of estrogen-receptor complex in human breast tumour cytosol. *Cancer Res.*, **40**, 2941–2942.

ROY, E.J. & MCEWEN, B.S. (1977). An exchange assay for estrogen receptors in cell nuclei of the adult rat brain. *Steroids*, **30**, 657–669.

SCATCHARD, G. (1949). The attractions of proteins for small molecules and ions. *Ann. N.Y. Acad. Sci.*, **51**, 660–672.